# Developments of a centimeter-level precise muometric wireless navigation system (MuWNS-V) and its first demonstration using directional information from tracking detectors

**DOI:** 10.1038/s41598-024-57857-7

**Published:** 2024-03-31

**Authors:** Dezso Varga, Hiroyuki K. M. Tanaka

**Affiliations:** 1 The International Virtual Muography Institute, Global, Tokyo, Japan; 2 HUN-REN Wigner Research Centre for Physics, Budapest, Hungary; 3 The University of Tokyo, Tokyo, Japan

**Keywords:** Particle physics, Civil engineering, Natural hazards, Solid Earth sciences

## Abstract

Various positioning techniques such as Wi-Fi positioning system have been proposed to use in situations where satellite navigation is unavailable. One such system, the muometric positioning system (muPS), was invented for navigation which operates in locations where even radio waves cannot reach such as underwater or underground. muPS takes advantage of a key feature of its probe, cosmic-ray muons, which travel straightforwardly at almost a speed of light in vacuum regardless of the matter they traverse. Similar to other positioning techniques, muPS is a technique to determine the position of a client’s muPS receiver within the coordinate defined by reference detectors. This can be achieved either by using time-of-flight (ToF) or angle of arrival (AoA) measurements. The latter configuration (AoA), called the Vector-muPS has recently been invented and the present paper describes the developments of the first prototype of a vector muometric wireless navigation system (MuWNS-V) with this new vector-muPS concept and its demonstration. With MuWNS-V, the reference tracker and the receiver ran wirelessly with fully independent readout systems, and a positioning accuracy of 3.9 cm (RMS) has been achieved. We also evaluated the outcome of measuring continuous indoor localization of a moving receiver with this prototype. Our results indicated that further improvements in positioning accuracy will be attainable by acquiring higher angular resolution of the reference trackers. It is anticipated that “sub-cm level” navigation will be possible for muPS which could be applied to many situations such as future autonomous mobile robot operations.

## Introduction

Thus far, various indoor positioning systems have been invented and demonstrated. While Wi-Fi indoor positioning system (IPS)^1^ is commonly used, one example of the most successful state-of-the-art radio-wave techniques is Marvelmind Indoor “GPS”^2^ that enables a positioning accuracy of 2 cm which offers a similar level or better performance than GPS-RTK^3^. However, if there are radio frequency shielding obstacles such as metal or seawater between the access points and the client receiver devices, their navigation probes are blocked and as a result, the navigation quality will degrade significantly or the navigation signal itself will be unavailable. LiDAR^4^, Dead reckoning^5^, photographic-based imagery^6^ can also be used for specific purposes of navigation such as the ones used in the collision avoidance support system^7^, however, since these techniques do not provide coordinate values of the clients within the given coordinate, navigation routes cannot be programmed with these methods. On the other hand, navigation routes can be programmed by utilizing dead reckoning^5^. This is a method of estimating past and present positions based on the route traveled, distance traveled, starting point, drift, etc., and performing navigation based on that position information. An inertial measurement unit (IMU) is a type of a device used for dead reckoning navigation with inertial sensors (an integrated package of accelerometers and gyroscopes); the positioning update rate is usually high and the navigation accuracy is not affected by obstacles or other interference in the surrounding environments. However, even with an industrial level IMU, the positioning estimation error increases to more than 50 m within one minute mainly due to the gyroscope’s bias instability and angle random walk^8^. muPS was invented to address the aforementioned problems and to operate in situations where other positioning methods may fail or malfunction.

Cosmic-ray muons are elementary particles, created by natural phenomena in the upper atmosphere. Muons with energies much higher than natural radioactivity reach the Earth surface and subsurface which can be utilized for a number of possible applications including imagery^9–11^, navigation^12–14^, time metrology^15–17^, and cryptographic communication^18,19^. The main properties of cosmic muons have been known for decades, including precise information on their abundance (flux), energy distribution, and properties when interacting with material^20^. The muon flux can be well approximated with a $\cos (\Theta )^2$ dependence on the zenith angle $\Theta$, that is, at 45^∘^ there is still half as many muons as from directly above. The vertical flux is around 100–120 Hz/m^2^/s/sr on the surface.

A method to use cosmic-ray muons to determine the three-dimensional position of a receiver (muon detector), “muPS”, was introduced in 2020 by Tanaka^12^. For many applications, it is highly advantageous to have the option to use wireless, asynchronous communication between the reference system (in a known location), and the receiver (in an unknown location) for positioning. The apparatus system that enables muometric navigation is called the Muometric Wireless Navigation System (MuWNS)^13,14^. The concept of muPS or MuWNS relies on the fact that muons travel at nearly the speed of light. By measuring the relative arrival times between a receiver and multiple references, one can triangulate the position, similar to the satellite-based GPS systems. This version of muPS requires either (A) wires between the reference and the client’s receivers or (B) precise and stable clocks associated with the reference and the receivers. The restriction of (A) substantially degrades the flexibility of muPS operation, and that of (B) requires atomic clocks with extraordinarily high granularity, such as an optical pumping cesium clock, for stable and acceptable navigation accuracy levels (< 10 cm)^14^. For example, if it takes 10 s in order to collect four muon tracks for determining x, y, z, and t, granularity of these clocks must be better than 300 ps in 10 s. On the other hand, short-term stabilities (RMS) of a commercially available Rb chip scale atomic clock (CSAC) is 1 ns in 10 s^21^. These problems can be solved with Vector muPS, a concept which was proposed by Tanaka in 2023^22^ which additionally exploits directional information. The Vector-muPS concept consists of the following key elements:

- A Reference (with a known position and orientation) is equipped with a good angular resolution, high efficiency, and possibly large-scale (large aperture of the order of few square meters) muon tracking system. The tracker should capture muons over a sufficiently broad angular range, typically up to 45^∘^.
- A Receiver (with unknown position) is the device that will be positioned by the Reference. The principal objective is to determine the location of the Receiver with sufficient precision, within a practically convenient time, and to follow its motion if it is not stationary. The Receiver must have an angular acceptance range for muons which is compatible with the Reference.
- One can assume that there is some limited, wireless, non-synchronized communication channel between the Receiver and the Reference, e.g. WiFi, ultrasonic, infrared or light-based data transfer.

The present paper describes the full functionality of a MuWNS-V. This new system consists of a Reference (tracking detector) using four MWPC chambers^23^, as well as a Receiver (comprising four, smaller and simpler MWPC-s). The two systems run two independent readouts based on Raspberry-Pi microcomputers, components which have been proven to be an efficient and reliable solution for other established muography projects such as imaging the Sakurajima volcano^24^ and for measurement of various underground structures^25^. The detectors are described in more details in the “Methods” section.

A single muon provides only two dimensional information, whereas multiple muon updates give unambiguous 3D positioning as long as the trajectories are not parallel. For the current study, this natural extension from 2D to 3D was not investigated, but it was assumed that the height difference between the detectors is known.

Some measurement uncertainties arise from the statistical and physical properties of cosmic rays. Two unrelated particles may randomly hit the detectors, leading to a measurement background, which can be reduced by requesting sufficiently short time difference between the hits. Using directional information in the MuWNS-V concept, this background can be drastically reduced, since accidental coincidences will usually have different directions.

In the current work, results from two similar configurations are presented. The **“LowRes”** configuration applied a smaller Reference tracker with an angular resolution around 15 mrad, which resulted in a positional accuracy of 3.9 cm (RMS) with the Reference located 2.4 m above the Receiver. An improved version, which will be referred to as the **“HiRes”** configuration, was constructed using larger area Reference chambers, and the angular resolution was improved to 6–8 mrad (using the same Receiver tracker). Moreover, the detectors were located at two different floors of the building. The positioning accuracy was improved to better than 3 cm (RMS) at 3.3 m elevation difference between the detectors.

Both the Reference and the Receiver operated independent internal clocks. Whenever a muon crosses both detectors, called “updates”^22^, one can check if the detections took place within a certain time window. This is called the “verification time window”, $T_W$. That is, any pair of detected particles, be it a valid muon or a background (two random particles or a shower) with time difference below $T_W$ will be defined as updates. The frequency of these updates will be denoted by $f_U$.

## Results

### Experimental setup

**Figure 1:**
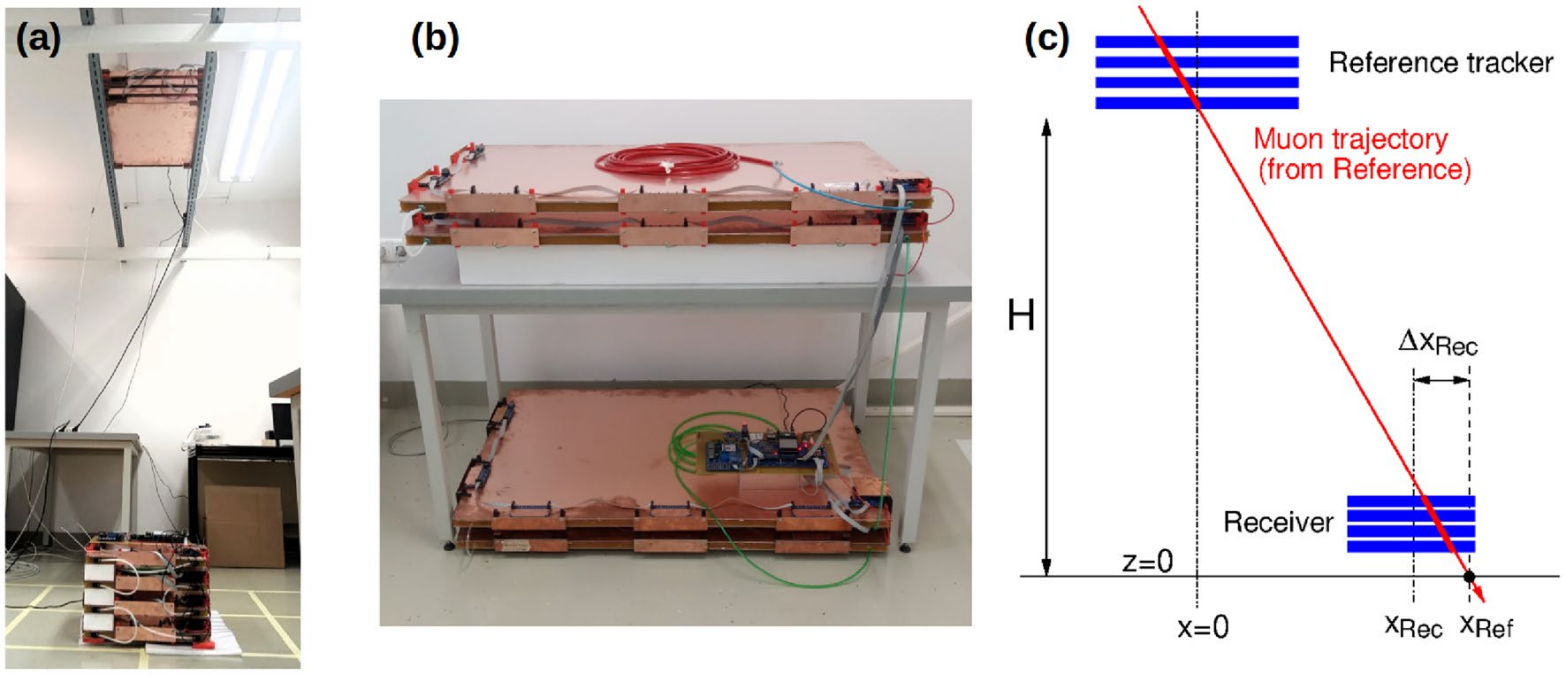
The experimental setup. (**a**) Photograph of the “LowRes” detector system, with Reference attached to rails below the ceiling, and the Receiver on the ground. (**b**) Reference tracker of the “HiRes” system. (**c**) Illustration showing the geometrical parameters.

The measurements were performed indoors, with the Reference tracker at $H = 2.4$ m or $H = 3.3$ m above the Receiver. Figure 1 shows a photograph of the detectors (both “LowRes” and “HiRes” configurations), along with an illustration to clarify the geometrical parameters. The sensitive total area (for muons passing vertically through the horizontal chambers) of the Reference was $S_{Ref}$ = 0.25 m^2^ in the LowRes, and $S_{Ref}$ = 0.86 m^2^ in the HiRes configuration. The Receiver area was $S_{Rec}$ =0.145 m^2^. The Receiver was moved in *x* and *y* directions, at a few elevations with a fixed *z* value.

The current experimental setup aims to directly demonstrate practical muometric positioning, improving the accuracy and applicability compared to conventional muPS^12^ which uses only time-of-flight and lacks directional information:

(A) A high precision and robust tracking system considerably improved positioning accuracy without requiring atomic clocks^26^.

(B) Cosmic muon rate is naturally limited to an order of 100 Hz/m^2^, which results in more than 1 ms between subsequent events. Muons which cross both detectors can be tagged without precise clocks or fast wireless communication ($T_W$ is practically in the order 0.1 ms)^22^ to ensure, and continuously maintain time synchronization between the Reference and the Receiver. This concept called “Cosmic Timing Calibration” (CTC)^17^ and removes the requirement to use wires and expensive clocks from the muPS system. In this work, the CTC-based synchronization is maintained, or in other words, the two detectors are “CTC-locked” to each other.

**Figure 2:**
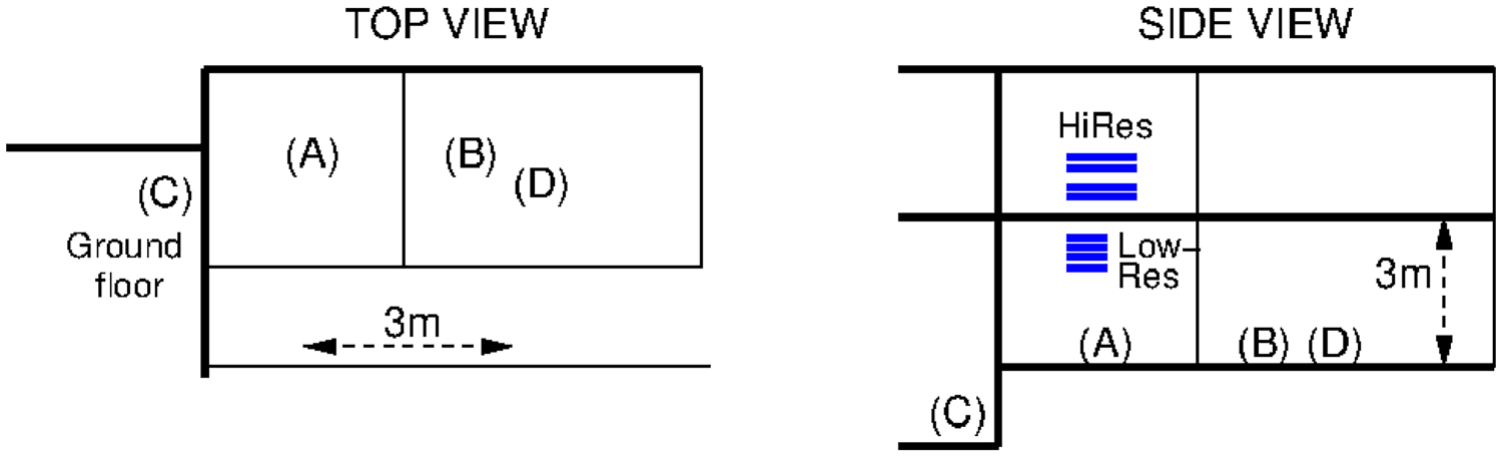
Geometrical configuration of the measurements inside the building. Letters indicate the Receiver positions. Horizontal bars indicate the Reference tracker chamber stacks. Lines correspond to the building walls and floors.

The measurements were performed indoors, such that the Receiver was always close to the floor. In the LowRes configuration, the Reference tracker was indside the same room, on rails close to the ceiling. For HiRes measurements, the Reference was moved to the upper floor, thus a 30–40 cm thick concrete structure separated the trackers. The geometry is illustrated in Fig. 2. Here thin lines indicate walls between rooms, whereas thicker lines correspond to 40 cm thick load-bearing walls and floors. For example, muons to reach position (C) needed to cross a concrete floor as well as a thick wall after the Reference.

### Positioning error of a stationary receiver

In the experiment, both the Reference tracker and the Receiver were MWPC-based detector stacks, capable of determining the muon trajectory with a certain precision. With both detectors, the muon trajectory was projected to the floor ($z=0$ plane) in order to determine the horizontal (*x*, *y*) coordinates. The projected position from the fixed Reference is denoted by $(x_{Ref}, y_{Ref})$. One aims at estimating the unknown Receiver center position $(x_{Rec}, y_{Rec})$ with this configuration. The muon trajectory recorded in the Receiver intersects the $z=0$ plane at $(\Delta x_{Rec}, \Delta y_{Rec})$, measured relative to the Receiver center position (see Fig. 1c).

Let us assume that it is possible to identify when a muon passes through both the Reference and the Receiver (referred to as “updates” defined above). In this case, one can estimate the Receiver center position using the difference value between the projections:

1 $$\begin{aligned} (x_{Rec}, y_{Rec})= (x_{Ref}, y_{Ref}) - (\Delta x_{Rec}, \Delta y_{Rec}) \end{aligned}$$

The error of this position estimation is entirely dominated by the angular resolution of the Reference tracker, given the position resolution of both detectors is well below 1 cm.

**Figure 3:**
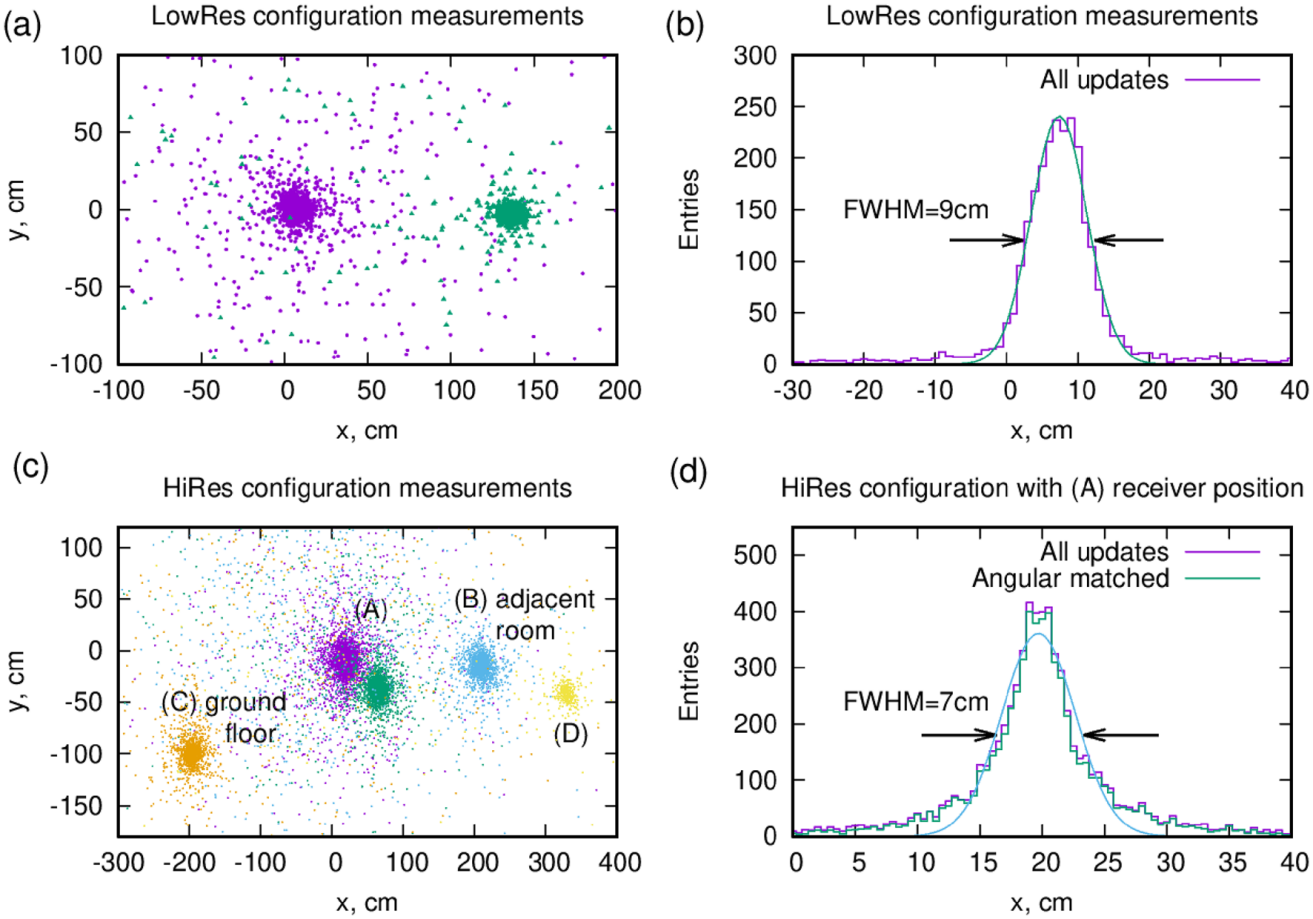
Measured position resolution. (**a**) Scatter plot of the estimated center points of the Receiver for all updates in LowRes configuration, at $T_W=0.1 ms$. (**b**) Distribution of the estimated Receiver positions $x_{Rec}$. (**c**,**d**) are corresponding figures for HiRes configuration.

Figure 3a,c show the scatter plot of the individual updates for various Receiver positions. With some background, the updates cluster around the fixed Receiver position. The position measurement error is 9 cm FWHM (3.9 cm RMS) in the LowRes configuration, and 7cm FWHM (3.0 cm RMS) in the HiRes configuration, as shown in Fig. 3b,d. (The Receiver was not centered, there is an arbitrary offset of the horizontal position.)

A summary of the quantitative results is presented in Table 1. The key parameters of a given configuration include the height difference *H*, the average zenith angle $\Theta$ of the matched tracks, as well as the distance of the detectors $D=H/\cos {\Theta }$. There is a notable non-Gaussian tail observable on the HiRes configuration, Figure 3d, due to the fact that the muons had to cross the ceiling, which induces scattering. One expects that this reduces the achievable position resolution, which is confirmed by the measurements. One can evaluate the expected position resolution based on the properties of the detectors (discussed in the “Methods” section), which is indeed lower than those observed.

**Table 1:** Summary of stationary measurement results: position resolution (RMS) and update frequency $f_U$.

Configuration (receiver pos.)	H (m)	$\Theta$ (^∘^)	D (m)	Reference angular resolution (mrad)	Expected pos. res. (cm)	Measured pos. res. (cm)	Expected, $f_U$ (Hz)	Measured, $f_U$ (Hz)
LowRes (A)	2.4	2	2.4	14.7	3.5	3.90	0.52	0.54
HiRes (A)	3.3	6	3.3	6.2	2.1	2.85	0.54	0.47
HiRes (B)	3.3	30	3.8	6.2	2.1	3.35	0.078	0.083
HiRes (C)	4.9	18	5.2	6.2	3.0	5.01	0.079	0.070
HiRes (D)	3.3	46	4.7	8.2	2.7	3.93	0.041	0.043

Depending on the Reference tracker angular resolution, the position resolution can be predicted. See text for explanation on update frequency.

### Update frequency: measurement and prediction

The update frequency $f_U$ is by definition the number of muons which can be confirmed to cross both Reference and Receiver, divided by the measurement time. Values of $f_U$ are indicated in Table 1. One can predict the update frequency based on the cosmic muon flux in the following way: The muon flux $\phi$ is related to the detected muon rate *R* from a solid angle region $\Delta \Omega$ according to the formula^20^:

2 $$\begin{aligned} R/(\Delta \Omega ) = \phi ({\underline{\theta }}) S ({\underline{\theta }}) \end{aligned}$$

where all the quantities, including the effective detector area $S({\underline{\theta }})$, depend on the direction vector ${\underline{\theta }}$. Let us assume that the Receiver is placed below the Reference tracker, then the update frequency $f_U$ can be estimated as

3 $$\begin{aligned} f_U = \phi _{Rec} S_{Rec} \Delta \Omega _{Ref} \end{aligned}$$

where $\Delta \Omega _{Ref}$ is the viewing solid angle of the Reference, seen from the position of the Receiver. This latter quantity can be approximated by

4 $$\begin{aligned} \Delta \Omega _{Ref} \approx S_{Ref}/D^2 \end{aligned}$$

since muons follow a straight trajectory ensuring the direction ${\underline{\theta }}$ is the same for both detectors. The update frequency can be estimated as

5 $$\begin{aligned} f_U \approx \phi _{Rec} S_{Rec} S_{Ref}/D^2 = (R_{Rec}/{\Delta \Omega }) (R_{Ref}/{\Delta \Omega }) /({\phi _{Ref}}D^2) \end{aligned}$$

The second form of $f_U$ is particularly convenient since it directly uses the measurable track rates $R/\Delta \Omega$. The muon flux at the Reference $\phi _{Ref}$ can either be readily estimated or measured, such as for the present paper, $\phi \approx 100 \cos ^2(\Theta )$ m^-2^ s^-1^ for the thin roof of the building. In case that the Receiver is underground so that the flux $\phi _{Rec}$ (and accordingly $R_{Rec}$) is attenuated, this formula holds.

The measured and estimated quantities have considerable errors, the estimated $f_U$ can have 20% uncertainty including all systematics. Considering this, the agreement is reasonable for the various measurement conditions.

### Positioning a moving receiver

**Figure 4:**
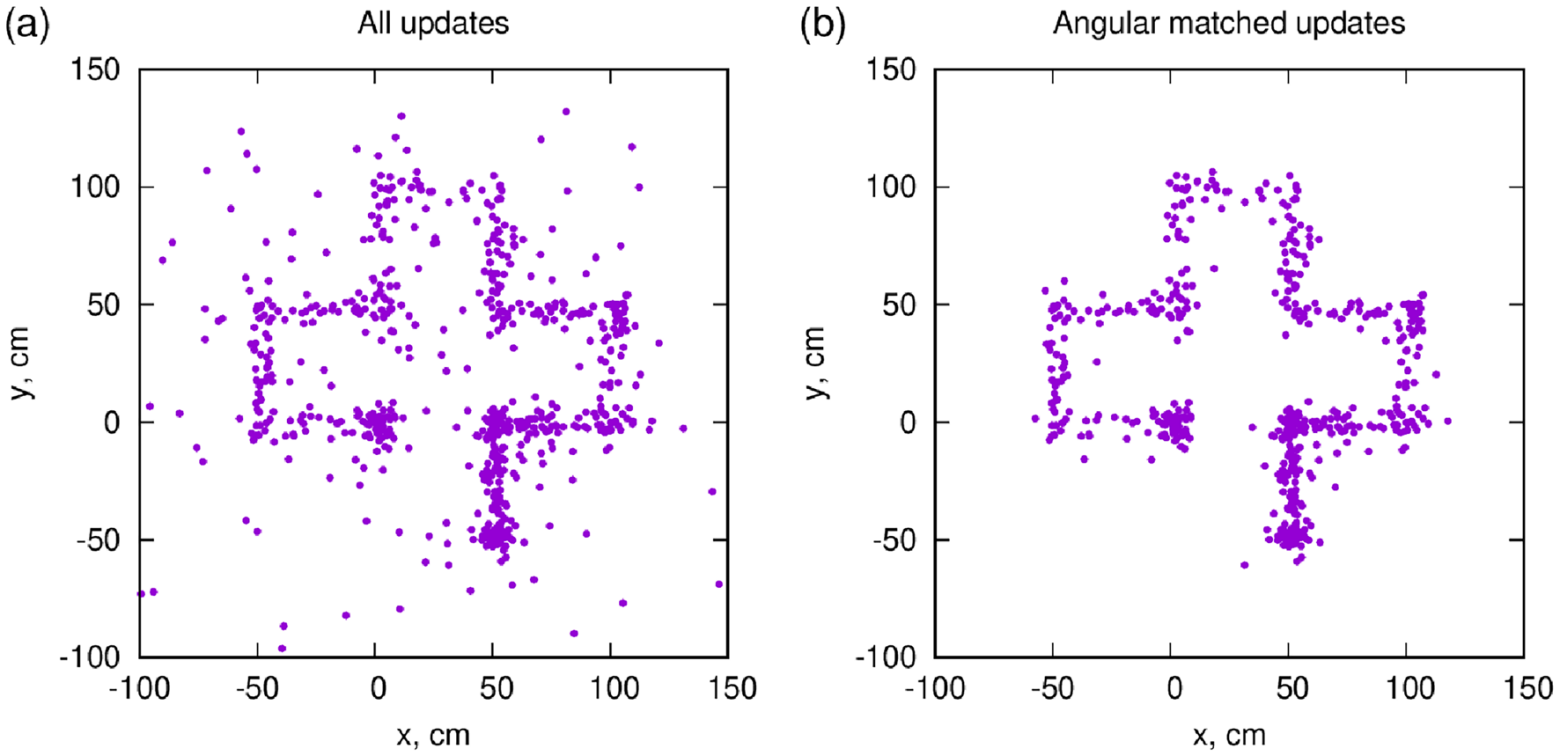
Dynamic measurement done by moving the Receiver horizontally along grid lines on the floor. (**a**) Including all updates, some background is apparent, which is suppressed requiring a 80 mrad angular matching (**b**) between receiver and reference.

When the Receiver is moving, in this experiment on the $z=0$ plane of the floor in the LowRes configuration, the updates continuously follow the position. For Fig. 4, the Receiver was moved slowly along grid lines spaced 0.5 m apart. The pattern of the motion, starting from the origin, is clearly visible. The total path was 5 m long, taken in about 25 min. Figure 4a shows all the updates, with some background scattered around the real path. Figure 4b shows the same data, but requiring an angular matching between Receiver and Reference, as explained in the next section.

### Background rejection using the direction information from the receiver

**Figure 5:**
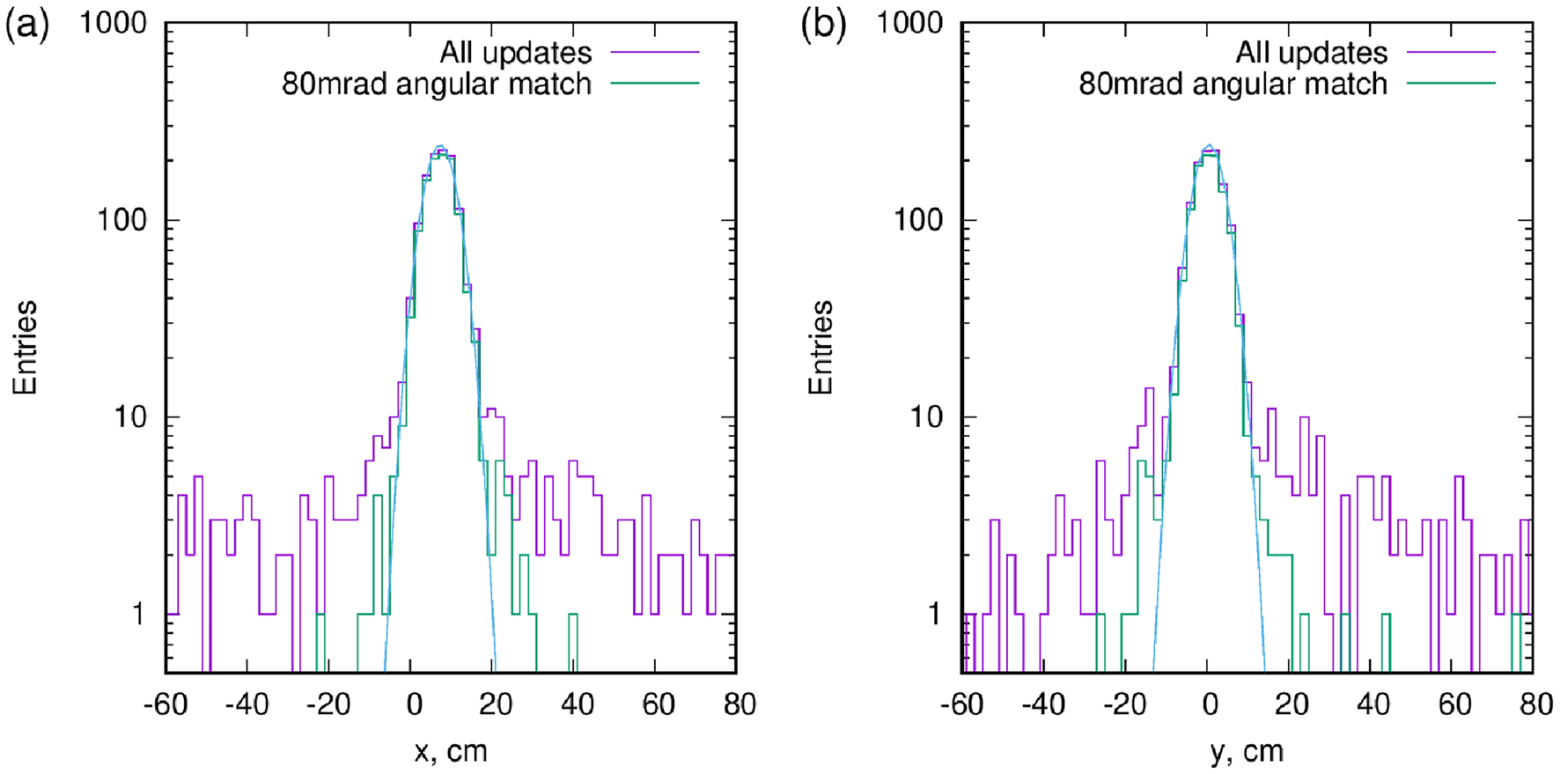
Distribution of the positions from the updates, with or without using angular matching between Receiver and Reference tracker. The tails apparent for all updates get strongly suppressed when restricting the orientation in parallel directions, within 80 mrad. Panels (**a**) and (**b**) show the x and y directions respectively.

In a general muPS configuration, the Receiver is a relatively small object, capable of only providing timing information. However, if the Receiver can measure a rough trajectory direction, the background may be reduced drastically^22^. If one requests that the Reference and Receiver trajectories are parallel to each other within an 80 mrad (4.6^∘^) window, most background vanishes, as demonstrated in Fig. 4b. The core of the distribution of the measured positions is unchanged, but the background populating the tails of the distribution is suppressed, as clearly apparent in Fig. 5. Quantitative estimation of this random background is rather complicated as it originates from air showers initiated by both primary cosmic rays as well as muon decays and other soft particles^27^.

### Improvement of positioning accuracy by averaging

**Figure 6:**
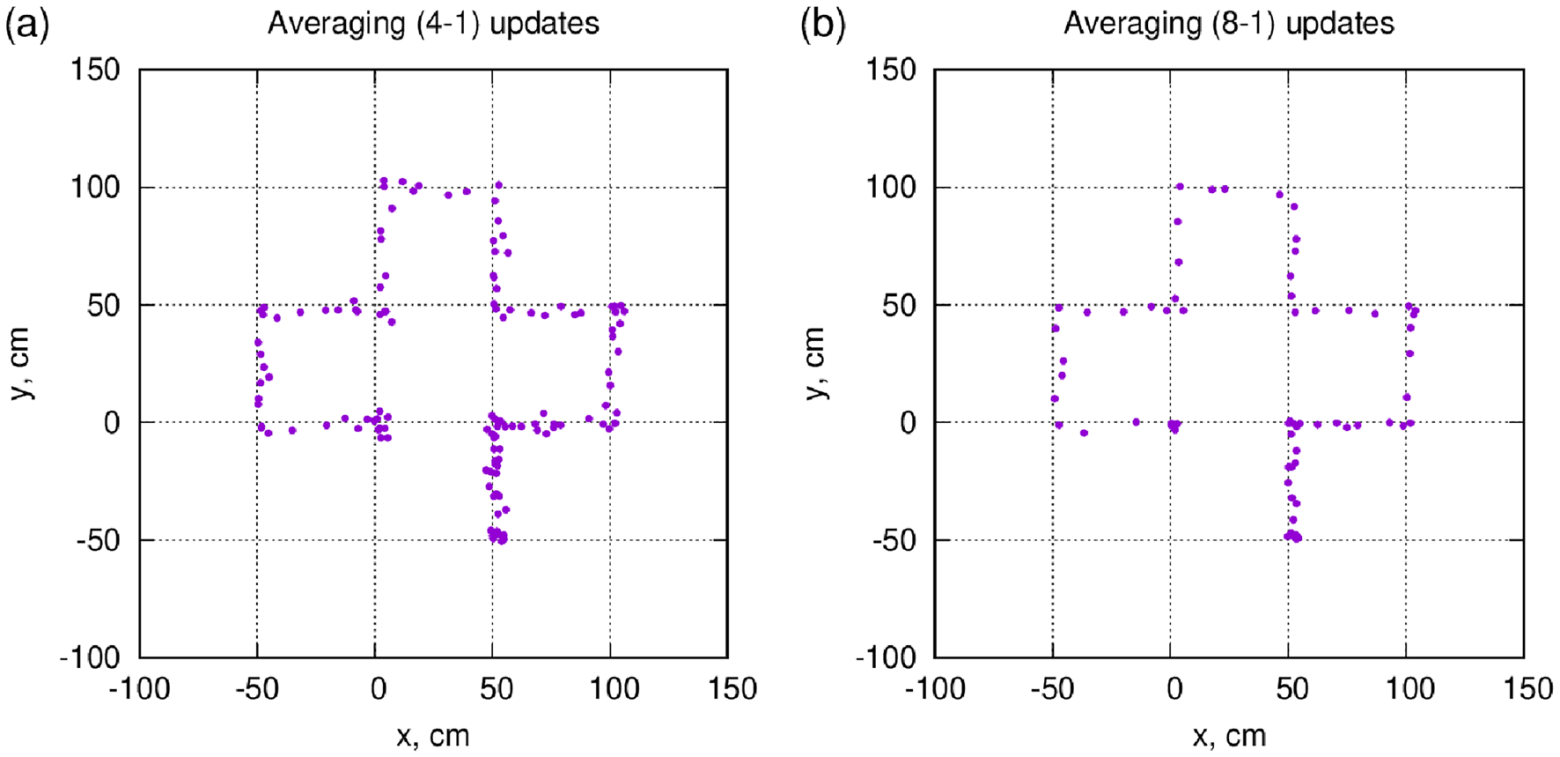
Improvement of positioning accuracy by averaging over multiple updates. (**a**) averaging over “4-1” measurement points, that is, eliminating one out of 4 subsequent updates which are the furthest from the average. (**b**) the “8-1” average.

Since the directional muPS concept is statistical by nature, averaging over multiple update measurements gradually improves the positioning accuracy. This is demonstrated in Fig. 6, which can be compared to Fig. 4. A simple and consistent method to remove background outliers is to truncate the averaging: for Fig. 6, one takes *N* subsequent measurements, then takes the average, and then drops the one which is the farthest from the average. Subsequently, the remaining $N-1$ are averaged. Figure 6a shows $N=4$ and Fig. 6b for $N=8$, where the positioning accuracy improvement is clearly visible, which is consistent with the $(N-1)^{-1/2}$ rule expectation.

### Maintaining the CTC lock

Given the fact that the Receiver can move or be anywhere, the only means to ensure that one can tag muons which cross both trackers (that is, a means of finding the updates) is sufficiently precise timing information. The readout systems of the two detectors are fully independent, therefore some synchronization must be achieved.

Precise time synchronization can be naturally achieved using the CTC concept^17^, as explained in the Introduction. Updates are not only useable for measuring position, but at the same time, can actively adjust the internal clocks. This method can maintain the synchronization continuously, that is, the two systems are “locked” to each other. Once this CTC lock is captured, there is negligible chance to lose it (due to a sufficiently low level of random background).

For the measurement system presented above, two clock types were used: the low precision internal clocks of the two Raspberry-Pi microcomputers, as well as a Temperature Controlled crystal ocillator chip (TXCO, IQXT-200-49 series) with a rated precision of 0.05ppm. This can be further improved using oven controlled crystal oscillator (OCXO)^14^, however the TXCO precision proved sufficient for the current studies.

**Figure 7:**
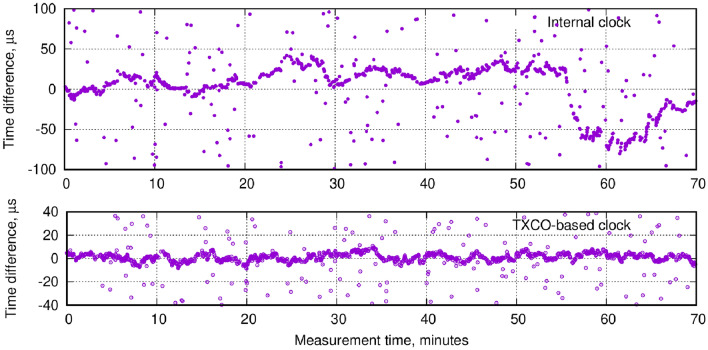
CTC lock in practice. The vertical scale shows the time difference between a trigger time stamp in Receiver and Reference. The accumulation of points around zero corresponds to updates, whereas scattered points represent random background. The top panel shows the case using only the RPi internal clock, whereas the bottom panel shows the improvement by a TCXO clock.

Figure 7 shows how the “CTC lock” is maintained, for a time period of more than one hour. In the LowRes configuration using the internal clock of the Raspberry-Pi computers, shown on the top panel, the update frequency was 0.31 Hz—that is, updates happen on average 3 s apart. After capturing 8 updates within the verification time window $T_W$ the Receiver clock is (off-line) adjusted to the Reference clock. The time difference between updates apparently drifts by 10–20 $\mu$s on the scale of minutes, which amounts to an order of a few ppm. Using the TCXO clock in the HiRes configuration, shown on the bottom panel of Fig. 7, the time drift is much smaller as expected.

One must note that smart algorithms such as machine learning can considerably improve the verification of updates, by optimizing the conservative $T_W$ value, and exploiting constraints on the position of the Receiver (background usually appears far away from the real position). In addition, for Fig. 7, angular matching was not used.

### Capturing the CTC lock

It has been demonstrated that once updates are verified, all subsequent updates can be captured to follow the motion and maintain synchronization. The question remains, how does one verify the first (few) updates unambiguously? In a possible practical configuration, such as an event of emergency, the Receiver is switched on such that its clock can be totally off from reality (by seconds), and its position is unknown. Below this essential function is demonstrated for the measurements in the present paper. Standard WiFi-like connection can easily adjust the clocks on the order of 0.1 seconds, whereas $T_W$ = 0.1 ms is fully sufficient to maintain the CTC lock; one must span this to 3 orders of magnitude in precision.

**Figure 8:**
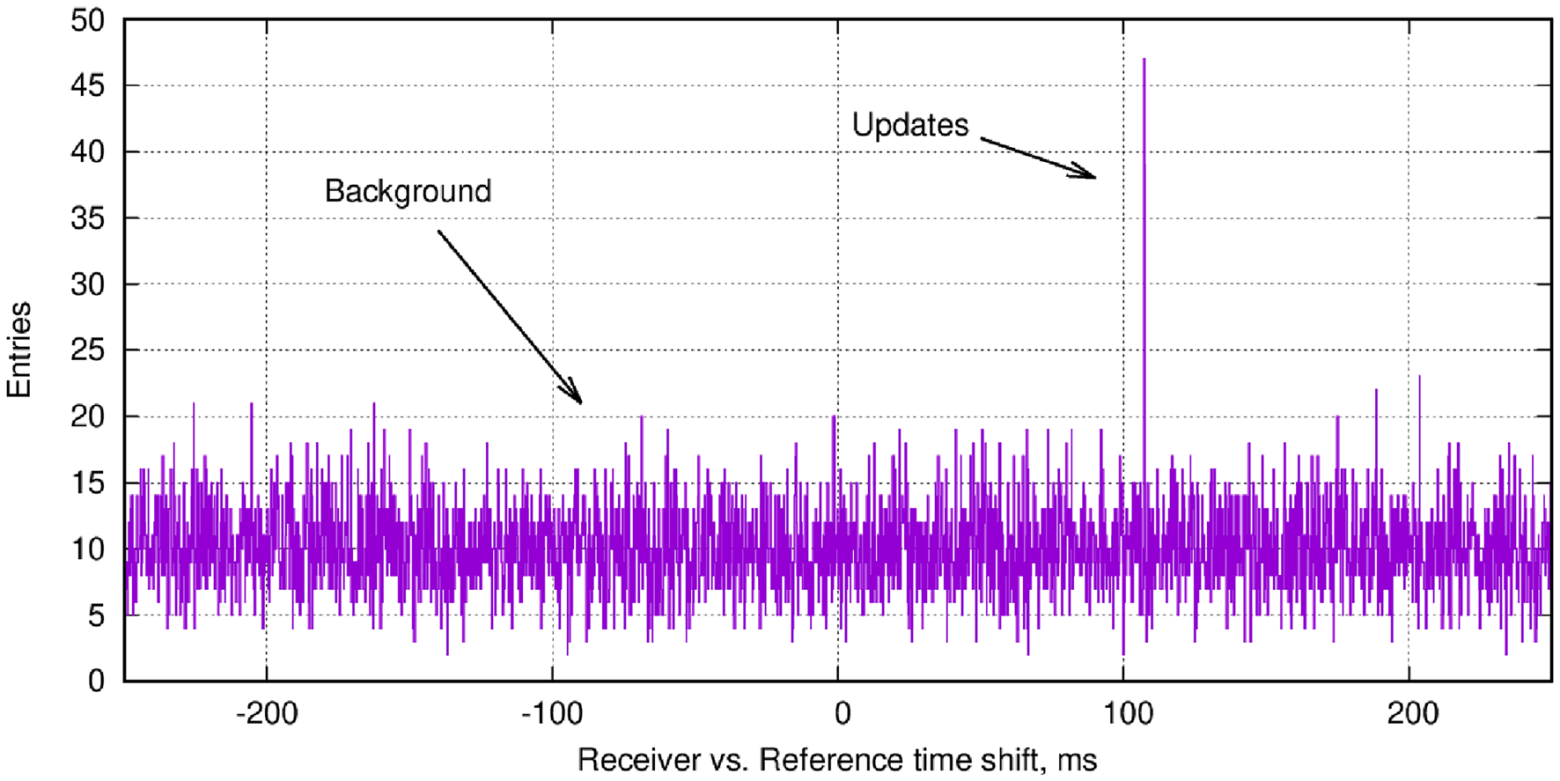
As a function of the time shift $\Delta t$ between Receiver and Reference time stamps, the vertical axis shows the number of cases when the time difference is smaller than the verification time window $T_W$ between any combination of events (see text for clarification). The sharp peak corresponds to the real updates. All other values are from random background. Data taking time was 2 min.

In the first step, both Receiver and Reference should take data for a few minutes (10–100 updates). Then the Receiver shares the time stamps with Reference, resulting in a time sequence $T_{Rec,1} \dots T_{Rec,N}$ . Accordingly, the time sequence for Reference is $T_{Ref,1} \dots T_{Ref,M}$ (both N and M, that is, the total number of events recorded, are in the order of $10^4$ – $10^5$). One can then evaluate for all combinations (*n*, *m*) the time differences $t_{diff}= (T_{Rec,n} - T_{Ref,m})$. The next step is to count cases when $-T_W<(t_{diff}-\Delta t)< T_W$ . $\Delta t$ represents the unknown initial shift between the clocks, covering a typical range of 0.1–1 s, for $\Delta t$ in steps of $2 T_W$. One expects a peak corresponding to the true $\Delta t$ value between Reference and Receiver: in that case, the true updates are adding up, whereas when $\Delta t$ is not the correct value, one gets only random (background) combinations. A typical such “time correlation” plot of the time sequences is shown in Fig. 8, using data taken for only 2 minutes, at update frequency of 0.5 Hz. In that case, the true time shift between the clocks was around 110 ms. With this information, which is precise within an error of not more than $T_W$, the independent clocks can be initially adjusted. From then on, the CTC lock synchronization can be maintained for an arbitrarily long time.

## Discussion

The most recent iteration (current work) of MuWNS-V has two main elements (Reference and Receiver); Reference (a cosmic muon tracker with a known position) points towards a smaller, possibly less complex detector called Receiver. Every time a muon crosses both Reference and Receiver, the muPS PNT (positioning, navigation and timing) information is “updated” for the Receiver position and time synchronization between Reference and Receiver. A key point of the MuWNS-V is that Receiver and Reference can be strongly synchronized with a relatively slow wireless connection by using the CTC lock. With this CTC locking technique, even if the internal clocks of Receiver and Reference are not synchronized right after switching on these clocks, within the first several coincidence events, these two clocks are eventually synchronized by using muons that cross both Reference and Receiver. This approach is similar to the standard GPS systems, which require a few minutes (Time To First Fix) to give precise and continuous position information.

In the current work, it was shown that CTC locking was achievable after capturing 10–100 coincidence events, which eliminates the need for highly sophisticated timing solutions. The results of the most recent experiment have demonstrated full operability of MuWNS-V under such conditions. Although accidental coincidence events can cause mis-positioning, this can be resolved by taking angular coincidence measurements between tracks at Reference and those at Receiver. MuWNS-V is most effective in situations when there is some connection (WiFi, Bluetooth, ultrasonic, etc.) available, but the position of the Receiver is unknown (e.g. GPS is unavailable). As described before, this may happen in underground or underwater conditions, or in a large building indoors separated by walls, or in an emergency rescue situation with some overburden. If a precise 3D positioning is required in a large volume (e.g. a whole building) then multiple Reference stations can be installed in a horizontal plane such that from any point of the volume of interest, update muons can be captured from a reasonably steep angle. In this case the Reference detectors are naturally larger and more precise than the mobile, lightweight Receivers.

It is often advantageous to use two techniques in tandem to get the maximum benefit from both methods and to more effectively improve accuracy with calibration: such benefits are possible by combining muPS and Wi-Fi indoor positioning system (IPS). It is predicted that the global market value of Wi-Fi IPS will expand to 19 billion dollars by 2030^1^. The clear benefit of Wi-Fi is, due to its widespread installation in buildings and its integration into smartphones, it can be nearly effortlessly retrofitted for the purpose of indoor positioning. The Wi-Fi positioning system (Wi-Fi IPS) offers indoor navigation capability for people with smartphones. Positioning accuracy of Wi-Fi IPS alone is limited to about 20 m without calibration; however, with calibration, Wi-Fi IPS can achieve 5–8 m accuracy. In order to attain further accuracy, various techniques such as Wi-Fi fingerprinting, ToF and AoA are combined to attain a sub-m positioning accuracy. Unlike other radio-wave-based IPSs, such as Bluetooth Low Energy (BLE), ZigBee, and Radio Frequency Identification (RFID), Wi-Fi IPS does not require additional infrastructure since many of buildings already equipped with Wi-Fi access. Therefore, it is more reasonable to choose Wi-Fi IPS when the required positioning accuracy is in the order of one meter. This caveat is more or less the same for muPS as well: we need to deploy reference detectors in addition to Wi-Fi access points. Nevertheless, it is unusual that cm level navigation accuracy is required throughout an entire building. In many cases, we only need this level of accuracy in a localized area such as the IPS calibration points, the wireless power transfer (WPT) station for charging the autonomous robots, the area where articulated robot arms are located, etc. Working together in tandem, muPS offers positioning accuracy that is not affected by the surrounding environment and Wi-Fi IPS offers direct, practical communication via smartphones. For example, even if we sparsely deploy the muPS references on the ceiling of the indoor space, Wi-Fi IPS can be automatically calibrated when the clients are located near the muPS reference.

**Figure 9:**
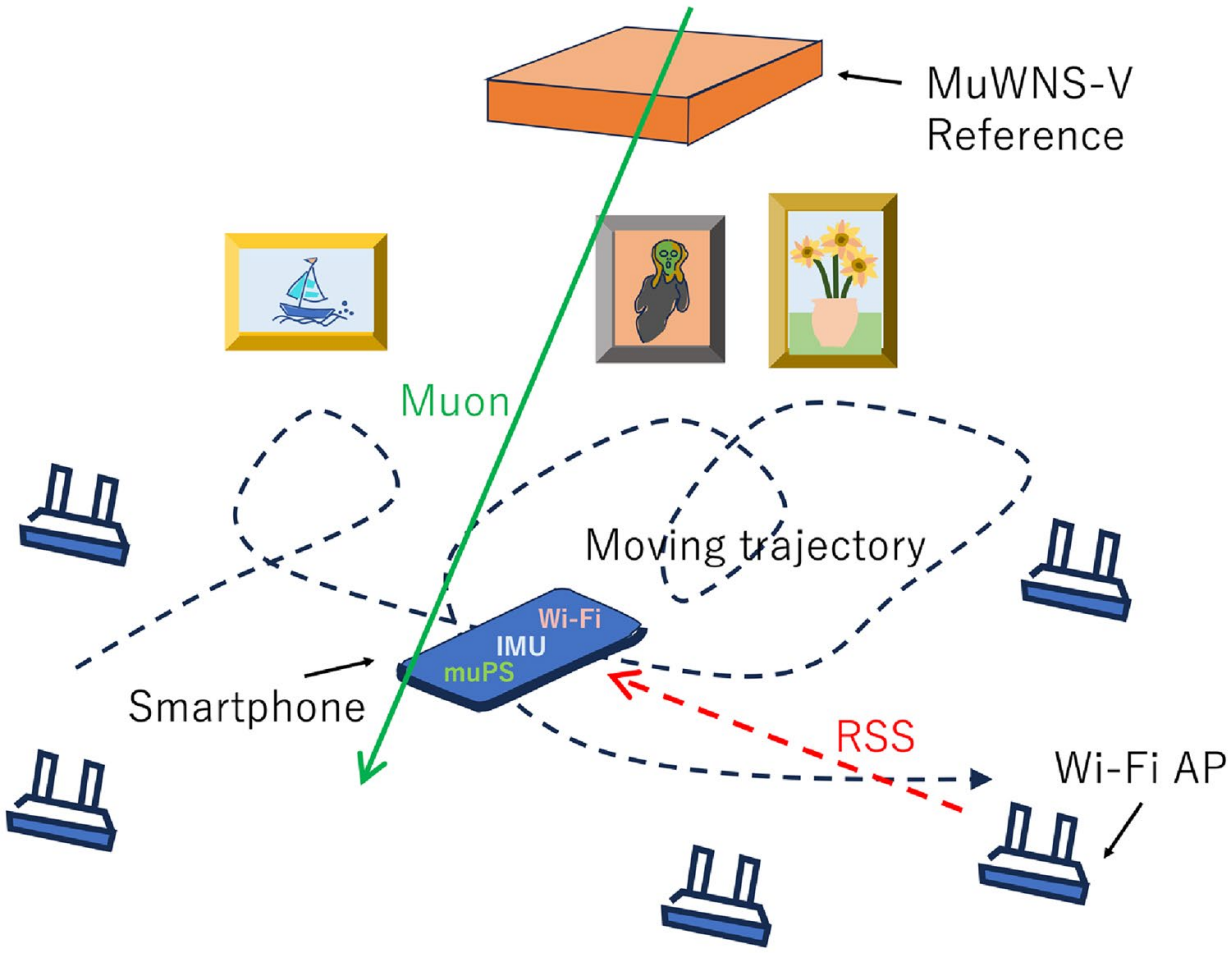
Wi-Fi-muPS hybrid navigation scheme in a museum. Wi-Fi IPS is repeatedly calibrated with MuWNS-V. RSS and IMU in this figure respectively stand for Received Signal Strength and Inertial Measurement Unit.

Unlike Wi-Fi IPS, clients cannot directly receive muPS signals with their smartphones in a practical way for direct utilization for real-time navigation. There are several reports claiming that muons could be detected by smartphone cameras by using the Complementary Metal-Oxide-Semiconductor’s (CMOS’s) silicon photodiode pixels to detect the photoelectric effect caused by muons^28^. The detection efficiency is relatively high ( 70%) so, the muon events can be logged, stored, and compared with the events detected by the muPS reference. However, the size of these smartphone chips is usually too small to attain sufficient muPS signal update rate to perform real time navigation by itself. Since the typical size of these chips is $\approx$ 1 cm^2^, the vertical muons would only be detectable at approximately the timescale of once per minute. Although this muPS signal update rate is too low for navigation, muPS on regular smartphones could be used for occasional calibration of Wi-Fi IPS. Thus, indoor navigation accuracy and convenience could be improved with this approach. Wi-Fi-muPS hybrid navigation scheme is shown in Fig. 9. In this use case, the clients are navigated inside a museum. Each location and each artwork are tied in the navigation program, and clients are navigated to each artwork by this program. If the clients stay in the exhibition room for 10 minutes, their IPS in their smartphones will be calibrated by using MuWNS-V about 10 times before they leave the room.

### MuWNS-V-IMU hybrid navigation system

In order to suppress the bias instability of the gyroscope and accelerometer as well as angle and velocity random walk errors, a ring laser gyroscope (RLG), a fiber optic gyroscope (FOG), or hemispherical resonator gyroscope (HRG) rather than a micro-electromechanical system (MEMS) gyroscopes are recommended. However, the weight of the device tends to be heavier when the former three options are employed. For example, the weight of MEMS is usually lighter than 500 g, and the lightest model weighs only 1 g. However, the former three options weigh more than 500 g, and this weight tends to increase as a function of the device stability improvement with some FOG models weighing almost 10 kg^8^. There is also a cost problem. The cheapest model of MEMS costs only 10 dollars but some FOG models cost 60,000 dollars; with the former option, gyroscope bias instability is more than 1000 deg h^-1^ while the latter option suppresses it to less than 0.01 deg h^-1^^8^. The situation with accelerometers is also more or less the same. The power consumption is also a large factor for practical navigation. While the power consumption of less accurate MEMS can be reduced to less than 100 mW, fancy FOG models require more than 20 W^8^. These factors make it very difficult to design a practical and stable stand-alone system for IMU navigation. MuWNS-V can be coupled with IMU in order to overcome the shortcomings of both methods. IMU short term drift can be repeatedly corrected by MuWNS-V, and a disadvantage of MuWNS-V (low positioning update rate) can be compensated by IMU.

### MuWNS-V security

An IMU-based navigation device is self-contained thus, it can resist the influence of external disturbances well. In short, the location of an IMU is not identifiable by devices outside the system. Therefore, its navigation information cannot be jammed or spoofed. This is the major advantage against Wi-Fi IPS. While Wi-Fi is a widespread and well-known technology, it is highly susceptible to being cracked. Consequently, if utilized for navigation, malicious attackers could easily manipulate the Wi-Fi positioning information and as a result clients could be navigated to completely different locations. MuWNS-V used by itself can also be spoofed relatively easily since it uses Wi-Fi to communicate between the reference and the receiver. Potential attackers would only have to crack the system and provide incorrect reference tracking information to the receiver to interfere with its operation. However, this problem could be solved by using MuWNS-V in conjunction with the Cosmic Coding and Transfer (COSMOCAT) technique^18,19^. COSMOCAT could enhance the security of MuWNS-V with true-random-number-based cryptographic keys (generated from the arrival times of detected muons) which would encode the reference tracking information; each instance of tracking information could be encoded with a unique key that can be shared (without a physical connection) with the authentic receiver using the same keys automatically generated in the COSMOCAT process. By using COSMOCAT, the MuWNS-V-IMU hybrid navigation system could be immune against the threat of nearly all external cyber-attacks.

In conclusion, a new MuWNS-V prototype which is completely independent from preexisting navigation systems has been developed and demonstrated in this work. Unlike radio waves, acoustic signals, or laser beams, muometric positioning accuracy is not influenced by obstacles in its surrounding environment. The current muPS signal update rate was 0.3–0.6 Hz, but it can be improved by using larger reference trackers. Also, the current muometric positioning accuracy is 3.9 cm (RMS) which merely comes from the geometrical configuration (angular resolution) of the reference trackers. Therefore, it is anticipated that the current MuWNS-V prototype has a further potential to evolve towards sub cm real time positioning accuracy by attaining a muPS signal update rate of > 1 Hz. This system was successfully developed and its performance has been confirmed to be sufficient for precise indoor navigation.

## Methods

**Figure 10:**
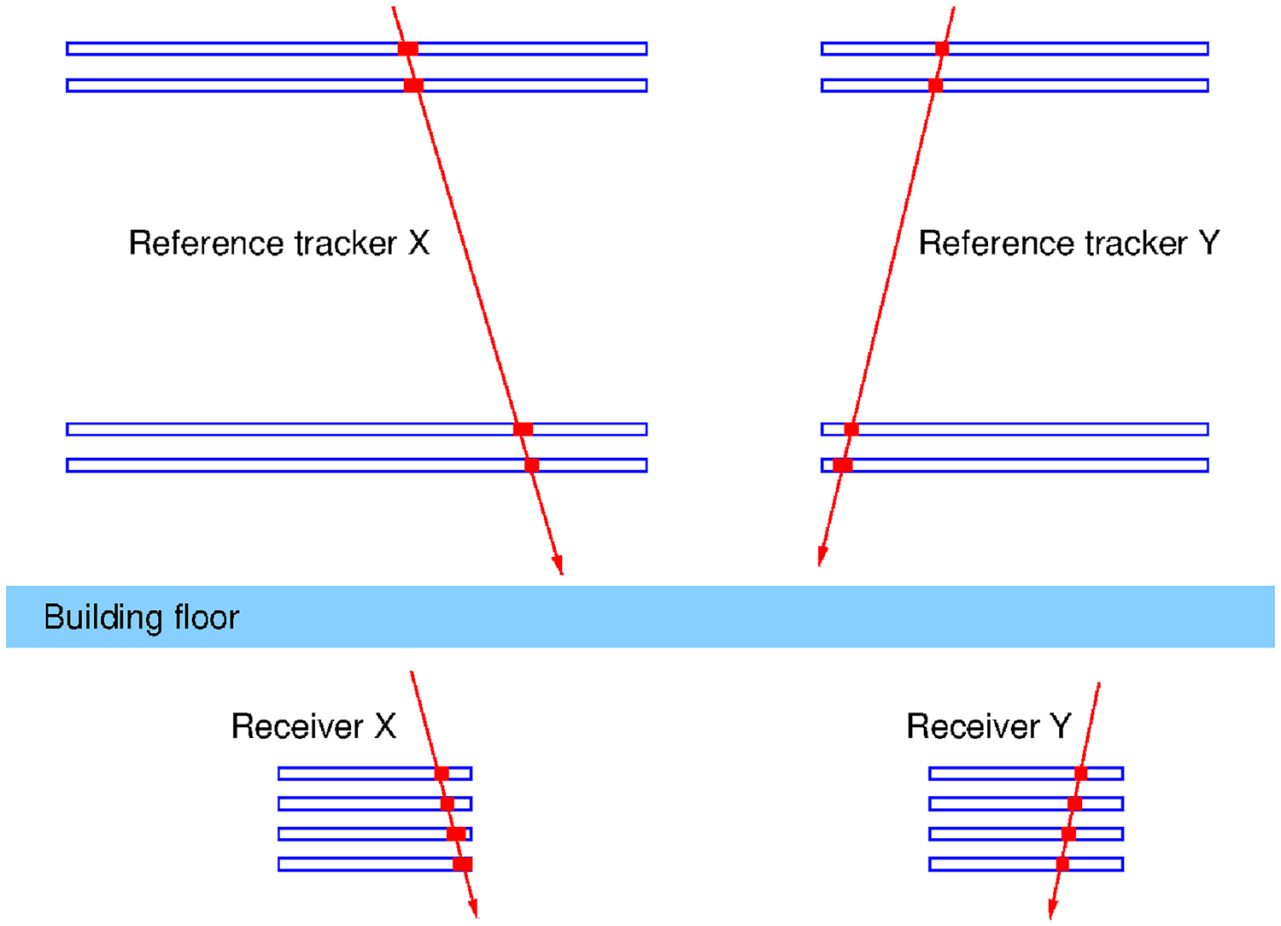
Example of an update: an actual event pair in the HiRes configuration, with Receiver at position (**A**). The detector relative geometries are approximately to scale. The Receiver was far lower than in this figure, close to the floor of the bottom room.

The tracking systems for the purpose of this demonstration were MWPC detectors^23^, similar to those which proved long term reliable operation capability at the Sakurajima Muography Observatory^24^. Four layers (chambers) were used for both trackers. The Receiver was a small, square shaped 40 cm detector stack with 12 mm segmentation, and 20 cm separation between top and bottom chambers. The Reference stations were larger: in case of “LowRes” configuration, 50 cm square chambers with 8 mm segmentation were applied with a top-bottom distance of only 17 cm. For the “HiRes” configuration, the Reference detector was much larger, 80 cm by 120 cm size with 12 mm segmentation, with as much as 1m total height. This larger separation led to a reasonably good angular resolution.

The detector geometry is illustrated in Fig. 10, for an actual matched pair of events, with the arrows corresponding to the estimated muon trajectories. The horizontal boxes represent the MWPC chambers, approximately to scale. One can note that in both directions (X and Y) the tracks are parallel, which confirms that indeed this was the same muon and not an uncorrelated background event.

**Figure 11:**
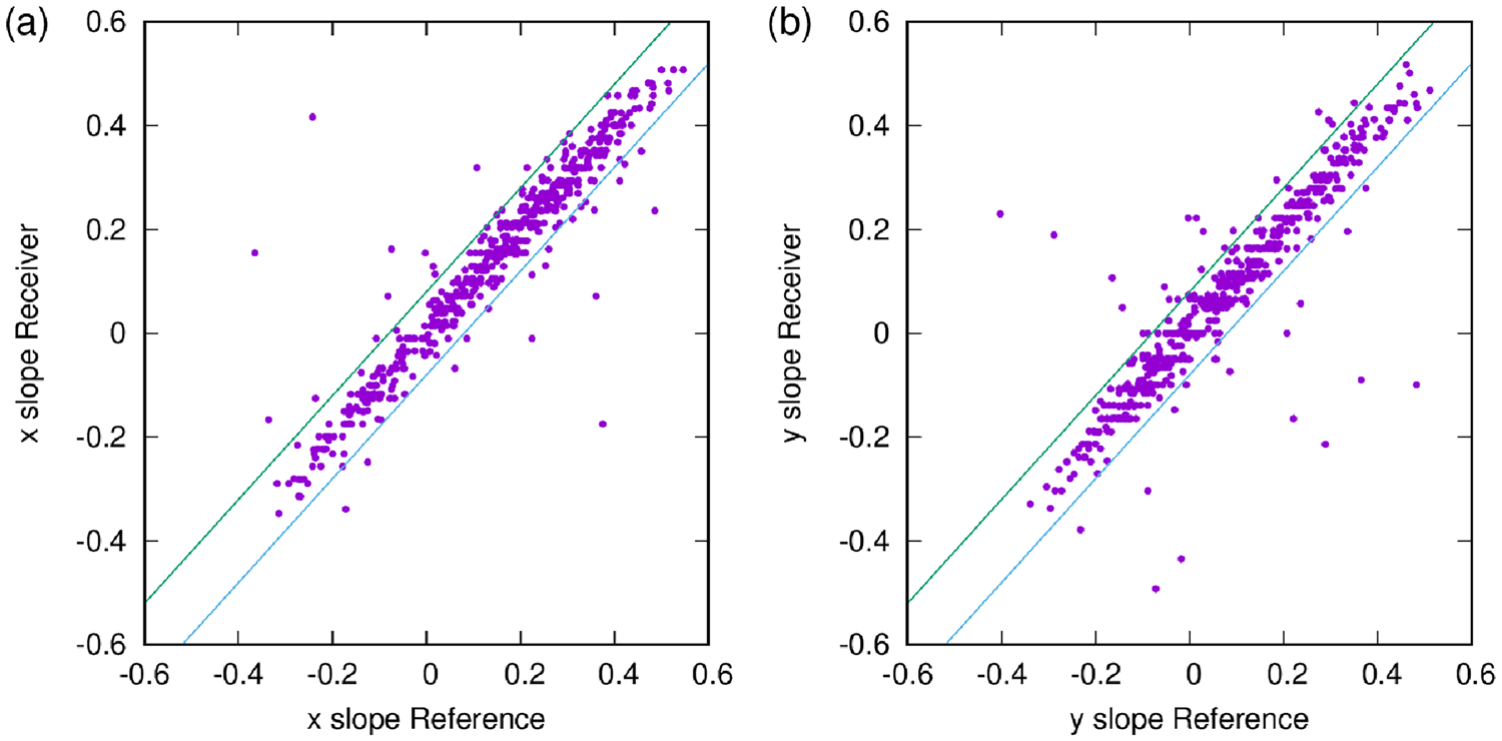
Angular matching between receiver and reference. The figure shows the slopes (tangent of the angles) in x and y directions. The lines indicate the ± 80 mrad angular window which defines the angular matching.

The angular matching (correlation of track directions) is shown in Fig. 11. The angular resolution of the Receiver is worse than that that of the Reference, however, 80 mrad captures most muons, and as it was demonstrated, helps in the task of reducing the random background.

The angular resolution of the Reference tracker can be estimated directly from the known position resolution^23^ and the spacing between the tracking chambers, indicated in Table 1. The position resolution of the navigation is expected to be the product of the Reference angular resolution and the *D* distance of Reference and Receiver.

The detector systems ran with fully independent Data Acquisition (DAQ) systems, which were based on a Raspberry Pi (model 3) microcomputer. The choice was motivated by its highly versatile nature, being similar to the capabilities of a standard smartphone, as well as its field-tested reliability. The two systems were taking data in parallel, but were started and stopped independently, and ensuring that there was no physical connection between the two - except for the cosmic muons.

The data contained individual muon trajectories, along with event time stamps. The intrinsic resolution of the TCXO-based clock was 200 ns, whereas the measured time difference between the timestamps of confirmed updates, including drift and detector resolution, was typically 1–3 $\upmu$s. It is important to note that the data transfer rate was considerably low. Since the information from a single muon is merely around 50 bytes, with the Receiver running at 20 Hz trigger rate, the necessary total data transfer rate is as low as 1 kbyte/s.

## Data Availability

The datasets used and/or analyzed during the current study are available from the corresponding author on reasonable request.
